# “I only seek treatment when I am ill”: experiences of hypertension and diabetes care among adults living with HIV in urban Tanzania

**DOI:** 10.1186/s12913-024-10688-8

**Published:** 2024-02-09

**Authors:** Theresia A. Ottaru, Christine V. Wood, Zeeshan Butt, Claudia Hawkins, Lisa R. Hirschhorn, Peter Karoli, Elizabeth H. Shayo, Emmy Metta, Pilly Chillo, Hellen Siril, Gideon P. Kwesigabo

**Affiliations:** 1 Department of Epidemiology and Biostatistics, Muhimbili University of Health and Allied Sciences, Dar es Salaam, Tanzania; 2 Department of Medical Social Sciences, Feinberg School of Medicine, Northwestern University, Chicago, IL USA; 3 Phreesia, Inc, New York, NY USA; 4 Department of Psychiatry and Behavioral Sciences, Feinberg School of Medicine, Northwestern University, Chicago, IL USA; 5 Feinberg School of Medicine, Robert J Havey Institute of Global Health, Northwestern University, Chicago, IL USA; 6 National Institute for Medical Research, Dar es Salaam, Tanzania; 7 Department of Behavioral Sciences, Muhimbli University of Health and Allied Sciences, Dar es Salaam, Tanzania; 8 Department of Internal Medicine, Muhimbili University of Health and Allied Sciences, Dar es Salaam, Tanzania; 9 Department of Psychiatry and Mental Health, Muhimbili University of Health and Allied Sciences, Dar es Salaam, Tanzania

**Keywords:** HIV, Cardiovascular diseases, Hypertension, Diabetes, Access to care, Qualitative research

## Abstract

**Background:**

For adults living with HIV (ALHIV) and comorbidities, access to comprehensive healthcare services is crucial to achieving optimal health outcomes. This study aims to describe lived experiences, challenges, and coping strategies for accessing care for hypertension and/or diabetes (HTN/DM) in HIV care and treatment clinics (CTCs) and other healthcare settings.

**Methodology:**

We conducted a qualitative study that employed a phenomenological approach between January and April 2022 using a semi-structured interview guide in six HIV CTCs in Dar es Salaam, Tanzania. We purposively recruited 33 ALHIV with HTN (*n* = 16), DM (*n* = 10), and both (*n* = 7). Thematic content analysis was guided by the 5As framework of access to care.

**Findings:**

The majority of the participants were females, between the ages of 54–73, and were recruited from regional referral hospitals. HIV CTCs at regional referral hospitals had more consistent provision of HTN screening services compared to those from district hospitals and health centers. Participants sought HTN/DM care at non-CTC health facilities due to the limited availability of such services at HIV CTCs. However, healthcare delivery for these conditions was perceived as unaccommodating and poorly coordinated. The need to attend multiple clinic appointments for the management of HTN/DM in addition to HIV care was perceived as frustrating, time-consuming, and financially burdensome. High costs of care and transportation, limited understanding of comorbidities, and the perceived complexity of HTN/DM care contributed to HTN/DM treatment discontinuity. As a means of coping, participants frequently monitored their own HTN/DM symptoms at home and utilized community pharmacies and dispensaries near their residences to check blood pressure and sugar levels and obtain medications. Participants expressed a preference for non-pharmaceutical approaches to comorbidity management such as lifestyle modification (preferred by young participants) and herbal therapies (preferred by older participants) because of concerns about side effects and perceived ineffectiveness of HTN/DM medications. Participants also preferred integrated care and focused patient education on multimorbidity management at HIV CTCs.

**Conclusion:**

Our findings highlight significant barriers to accessing HTN/DM care among ALHIV, mostly related to affordability, availability, and accessibility. Integration of NCD care into HIV CTCs, could greatly improve ALHIV health access and outcomes and align with patient preference.

**Supplementary Information:**

The online version contains supplementary material available at 10.1186/s12913-024-10688-8.

## Background

The establishment of HIV care services in Sub-Saharan Africa (SSA) has been highly successful [1, 2]. Over the past two decades, there has been remarkable progress in enhancing access to effective combinations of antiretroviral therapy (ART) regimens. Globally, in 2021, 79% of individuals living with HIV were accessing ART, which is a substantial increase from 31% in 2011 and 58% in 2016 [3]. ART coverage in Tanzania has also significantly improved; currently, among 1.6 million people living with HIV, 94% are on ART, a substantial increase from the 35% reported in 2013 [3, 4]. The improved accessibility of effective ART combinations has resulted in a significant reduction in AIDs-related morbidity and mortality [5, 6]. Adults living with HIV (ALHIV) are now living longer healthy lives, with a life expectancy that approaches that of the general population [7]. 

With improved life expectancy, new threats to health associated with age and HIV-related non-AIDS comorbidities have emerged [8]. ALHIV and adults without HIV infection are at risk for non-communicable diseases (NCDs) as part of the normal aging process. However, ALHIV have a disproportionately higher risk of NCDs because of persistent HIV-associated inflammation and immune activation, leading to arterial inflammation and atherosclerosis [9–11]. The excess risk is also mediated by other risk factors such as obesity and dyslipidemia that are related to prolonged ART use [11]. The emergence of NCD and NCD risk factors in the era of controlled HIV disease impacts negatively on the gains in controlling HIV and improving the quality of life of ALHIV [12]. 

Hypertension and diabetes (HTN/DM) are among the most common NCD risk factors among ALHIV contributing to the increased burden of cardiovascular disease (CVD) in this population [13]. In Tanzania, it is estimated that about 25–50% of ALHIV are hypertensive while 4–11% are diabetic [14–17]. In response, the Tanzanian national guidelines for the management of HIV introduced strategies to prevent and manage HTN and DM and consequently control the burden of CVD and other NCDs [18]. The guideline specifically recommends patient education, routine screening, diagnosis, and management of HTN, DM, and other CVD risk factors at the HIV care and treatment clinics (CTCs). Additionally, it recommends referring cases of persistent uncontrolled HTN/DM for further specialized management [18]. However, HIV CTCs in Tanzania are poorly equipped with trained providers, equipment, and medications to appropriately execute the guidelines [19, 20]. The HIV care and treatment program in Tanzania largely depends on donor funds, whose primary focus is on improving ART coverage and achieving viral load suppression among ALHIV and not HTN/DM nor NCD care. As a result, access to prevention, care, and treatment for DM and HTN among ALHIV in HIV CTCs remains largely inaccessible.

As the number of ALHIV with comorbidities such as HTN/DM increases, access to comprehensive and coordinated healthcare services for these conditions and related risk factors will be crucial for ALHIV to achieve optimal health outcomes. Currently, there is limited knowledge about the challenges and barriers faced by ALHIV in accessing healthcare services for HTN/DM in Tanzania both within HIV CTCs and externally. Previous research on access to HTN/DM care for ALHIV and integrated NCD care at HIV CTCs in Tanzania primarily addressed health system barriers, such as the lack of an NCD data capturing system and provider non-prioritization of NCD treatment at the HIV CTCs despite guidelines recommendations, with limited attention to patient experiences and perspective regarding accessibility of care for NCD comorbidities [20]. 

The Penchansky and Thomas’ 5As framework (availability, accommodation, affordability, acceptability, and accessibility), established in 1981, provides a holistic approach to identifying and addressing barriers to equitable access to healthcare services [21]. The framework is commonly used in healthcare research, policy, and practice to guide discussions and interventions related to improving access to healthcare services [22]. We used this framework in a qualitative study exploring ALHIV experiences and perceptions about accessing care for HTN/DM within HIV CTCs and other healthcare settings in Dar es Salaam. The evidence from this study may inform the integration of HIV/NCD care, proposing potential implementation strategies to improve care for ALHIV with comorbid conditions in Tanzania, SSA, and similar settings.

## Methods

### Study design

**We conducted a cross-sectional qualitative study employing a phenomenological approach to explore the lived experiences of ALHIV while accessing care for their comorbid HTN/DM both at their HIV CTCs and other healthcare settings.** Phenomenology was chosen because it provided an in-depth exploration of individuals’ experiences in specific situations; particularly the meanings, motives, and perceptions they assigned to that experience [23, 24]. In this study we adhered to the consolidated criteria for reporting qualitative research (COREQ): a 32-item checklist for interviews and focus groups (**Additional file 3**) [25].

### Study setting

This study was conducted in six high-volume HIV CTCs (defined as having ≥ 1500 registered clients) in Dar es Salaam, Tanzania. HIV CTCs are situated within the premises of a main health facility but function independently with their own operation and dedicated staff. HIV CTCs in this study represented different health facility levels including health centers (2), district hospitals (2), and regional referral hospitals (2). **All six HIV CTCs are government-owned public HIV clinics, receiving government human resources and infrastructure support. These clinics also receive assistance from an implementing partner, that supports ART supply, HIV viral load and CD4 testing, and provides equipment and additional human resources.**

Healthcare services at the HIV CTCs in Tanzania are provided free of charge for ALHIV. These include dispensing of ART, screening for opportunistic infections, HIV viral load monitoring, patient education, and counseling on HIV-related issues such as ART adherence. Clinic visits are scheduled monthly or every three or six months for stable patients. **In addition to HIV-related services, the Tanzania guidelines** [18] **recommend the provision of free-of-charge NCD-related care at the HIV CTCs.** These services include regular screening for NCD risk factors such as HTN and obesity (every visit), blood glucose monitoring (every six months), and lipids screening (yearly). Also, routine screening for DM symptoms such as polyuria, polydipsia, and polyphagia is recommended. HIV CTC providers are encouraged to also provide patient education promoting healthy lifestyle (healthy diet and physical exercise), prescribe and dispense HTN/DM medication (if available at the facility), and provide formal referrals to specialized health facilities for management of persistent uncontrolled HTN/DM. **Due to a lack of equipment and medication** [19] **NCD-related care is not routinely provided at the HIV CTCs.**

**When NCD-related services are sought outside the HIV CTCs, patients are required to pay out-of-pocket if they are not covered by health insurance**. At the district and regional referral hospitals, HTN/DM services are provided through NCD clinics which are located within the premises of the main facility. These clinics are equipped with internists with specialized training in NCD management. At the health center level, services are provided through outpatient clinics, which are equipped with general practitioners without specialized NCD training. Patients with persistent uncontrolled HTN/DM from health centers are referred to appropriate higher-level facilities for further evaluation and management.

### Participants and sampling strategy

Participants were purposively recruited from a parent study that assessed ideal cardiovascular health and CVD risk factors among ALHIV. Further details of this study have been published elsewhere [26]. Participants were eligible to participate if (1) aged ≥ 18 years, (2) had been in HIV care for ≥ 12 months prior to the start of the study (3) were found hypertensive and/or had diabetes in the parent study, (4) were aware of such diagnosis, and (5) had previously sought care for their HTN/DM at any health facility. To cover a range of experiences, we purposively selected participants with diverse characteristics of age, sex, type of comorbidity (HTN, diabetes, or both), and years lived with the comorbidity. Potential participants were contacted via telephone and were invited to participate by the HIV CTC nurse at their respective clinics and we first focused on participants with extreme recordings of blood pressure and RBG during the primary study. Those who agreed to participate were provided with a daytime interview appointment that was convenient for them. In the parent study participants were informed about the qualitative study and consented to be contacted if found eligible. Initially, we initially identified 30 participants based on Cresswell’s recommendations for sample size in phenomenological studies [24], however we reached data saturation at 33.

### Data collection

In-depth interviews were conducted among 33 ALHIV with HTN/DM using a semi-structured interview guide between January and April 2022. The guide was designed to gain information about participants’ experiences and perceptions about care for HTN/DM both at the HIV CTC and at another facility. The questions were centered on care for HTN/DM and extended to challenges faced throughout this process and the coping strategies employed. The interview guide was previously pilot tested among five ALHIV (two with HTN, one with DM, and two with both HTN and DM) at an HIV CTC with similar characteristics as the research setting and was adapted before data collection. The Lead author (TO) and one research assistant conducted the pilot testing. During piloting, we sought participants’ feedback on the tool, identified ambiguous and challenging questions (which were either simplified or removed), assessed the flow of the interview, and recorded the time taken to complete the questionnaire. These interviews were thematically analyzed to identify themes and gaps in information. The results of the pilot testing were discussed among the research team and the interview guide was revised accordingly to obtain the final version. After piloting, we developed a separate interview guide for participants who are currently not in any care for their HTN/DM (but had previously sought care for these conditions) to inquire about how they are managing their HTN/DM outside of the clinic. TO (female, epidemiologist) and other three research assistants (2 males and 1 female social scientists) sought informed consent and conducted in-depth one-to-one interviews (in a private room at the HIV CTC) with participants in Swahili language. TO (a PhD student conducting mixed-methods research on cardiovascular health and related care among ALHIV) and the research assistants have years of experience in undertaking semi-structured interviews.

During data collection, we monitored the information being collected, and the consistency of the emerging themes. Through this process, we identified other aspects that needed to be further explored in subsequent interviews. This process was continuous until we realized that the interviews generated no more new information and hence determined data saturation. Data analysis began immediately after the first few interviews and was part of the iterative process [23]. The interviews lasted 30–45 min, were audio-recorded, and field notes were taken to aid interpretation of the audio recordings. No repeat interviews were conducted. All participants in this study were compensated for their time.

### Data analysis

Audio recordings of the interviews were transcribed verbatim, translated into English, anonymized, and checked for accuracy. We used the qualitative research software program Dedoose [27]. Adopting a thematic content analysis approach [28], firstly, TO and PK independently read and re-read initial transcripts to get familiarized with the data. Recurrent issues regarding access to care for HTN/DM among ALHIV were identified, labeled, and used to generate initial codes through open coding. These codes were then compared, and disagreements were resolved by consensus through discussion with each other and the rest of the research team [29]. Agreed modifications to the codes were made accordingly. TO coded the rest of the transcripts using the final agreed codebook. Codes were thoroughly examined and relabeled, and similar codes were consolidated into categories. These categories were organized into themes using the 5As framework model. The framework has been previously used to describe the experiences of care including access challenges among individuals with chronic diseases such as diabetes [30], mental health [31] and HIV [32]. It consists of five access dimensions (Table 1) that were applied as a lens during our analysis and presentation of the findings. We applied a constant comparison approach to evaluate the efficacy of the codes and to create linkages within the data.

**Table 1:** The 5As access to care dimensions adapted for this study for ALHIV and hypertension and /or diabetes

Access dimension	Definition
Availability	The extent to which the providers or the health system provide services that meet the needs of the patients. In this study, we explored the availability of screening services and medication for HTN/DM for ALHIV.
Accommodation	The extent to which the services are organized to meet the constraints and preferences of the client. In this study, we explored how HTN/DM service delivery systems are organized to meet the needs of ALHIV with comorbidities.
Affordability	The extent to which the patients have the financial means to use the services. In this study, we explored the extent to which the patients can afford the cost of medication and medical consultations for the management of HTN/DM.
Acceptability	The extent to which the healthcare services provided are acceptable to the population they serve. In this study, we explored the patient’s attitude toward HTN/DM medication
Accessibility	The extent to which patients perceive the ease of physically reaching and using healthcare services. In this study, we explored the transport costs incurred by ALHIV to reach HTN/DM care services in addition to HIV services.

## Findings

### Characteristics of the study participants

Thirty-three ALHIV [median age of 56 years (range 34–75)] participated in this study. The majority were females (55%), between 54 and 73 years (55%), and were recruited from regional referral hospitals (52%). Among study participants, 16 had HTN, 10 had DM, and 7 had both HTN and DM. Four reported having additional chronic medical conditions (including dyslipidemia, ventricular hypertrophy, and cervical and prostate cancer). Most of the participants had lived with HTN/DM for more than two years (Table 2). Although all participants in this study had previously sought care for their HTN/DM at least once before the study, the majority (*n* = 23) were not currently attending any clinics for the management of their HTN/DM nor were being actively managed for these conditions.

**Table 2:** Characteristics of the study participants

Participants Characteristic	Number (*n* = 33)
Median age (range)	56 (34–75)
Age groups	*n* (%)
34–53	14 (42)
54–73	18 (55)
> 73	1 (3)
Sex	*n* (%)
Female	18 (55)
Male	15 (45)
Comorbidity type	*n* (%)
Hypertension	16 (49)
Diabetes	10 (30)
Both	7 (21)
Years lived with the condition	*n* (%)
<2 years	8 (24)
2–5 years	10 (30)
> 5years	15 (46)
Facility level	*n* (%)
Regional referral hospital	17 (52)
District hospital	9 (27)
Health center	7 (21)

### Overview of the qualitative categories and themes

We identified nine categories narrating the lived experiences of ALHIV while accessing care for comorbid HTN/DM in HIV CTCs and other healthcare settings and categorized them using the 5As framework, see Table 3. We identified two categories that outline coping strategies to manage the financial burden of medication, consultations, and transportation costs. **The participants’ perspectives on HTN/DM care were mostly consistent across different categories of participants’ characteristics (age, sex, comorbidity type, years lived with the condition, and facility level) and therefore were not presented separately across these categories. Individual participant perceptions are used to describe different themes on HTN/DM care access among ALHIV.** The following sections report on study findings quoting patients’ voices, additional quotes can be found in the **Additional file 2**.

**Table 3:** Codes, categories, and themes on the access to care for HTN/DM and coping strategies among ALHIV in Dar-es-Salaam, 2022

Themes	Categories	Code
**Availability**	Limited availability of screening services for HTN/DM at the HIV CTC	Inconsistency in BP measurement services at HIV CTCs
		Lack of diabetes screening at the HIV CTCs
	Lack of anti-hypertensives and diabetes medication at the HIV CTC	Advised to seek treatment (including medication) at other facilities
		HTN/DM medication not within HIV CTCs provided services
**Accommodation**	Perceived complexity of HTN/DM care at NCD clinics	Uncoordinated service delivery systems
		Uncoordinated appointment schedules
		Preference for HIV/NCD integrated care at the HIV CTCs
	Lack of (tailored) patient education that meets ALHIV comorbidities care needs	Inadequate patient education on HTN/DM management
		Contradicting patient education information on multimorbidity management
		Participant desire for comprehensive HTN/DM management discussions with healthcare providers
**Affordability**	Unaffordable cost of HTN/DM medication	Purchasing medication only when symptoms become overwhelming
		Inability to afford more comprehensive health insurance plans
		HTN/DM medication are not covered by the insurance plans they can afford
	Unaffordable consultation costs at HTN/DM clinics	Stopped medical visits due to consultation costs
**Acceptability**	Fear of side effects of HTN/DM medication	Fear of symptoms experienced after multiple medication use
		Fear of side effects believed to be caused by multiple medication use
		Fear of life-long use of multiple medications
	Perceived ineffectiveness of HTN/DM medication	Poor control of HTN/DM after a perceived long-term use of pharmaceutical medication
		Perceived improved HTN/DM control through herbal medication use
		Belief in alternative approaches (sleeping, drinking water, use of garlic) to control blood pressure
**Accessibility**	High transportation costs to the HTN/DM clinic	High transport costs related to the need to attend multiple clinics (NCD clinic and HIV CTC) scheduled on different dates within the same facility
		High transport costs related to the necessity of attending NCD clinics at a different facility from where the HIV CTC is located
**Coping strategies**	Adaptive coping strategies	Seeking financial assistance from family, community, and church members
		Reliance on lifestyle modification; physical exercise and balanced diet
		Reliance on lower-level facilities e.g., dispensaries, and community pharmacies near home for management
	Maladaptive coping strategies	Use of perceived cheaper herbal remedies to control HTN/DM
		Self-monitoring of symptoms at home, eliminating the need to visit health facilities
		Intermittent use of medication

#### Availability

##### Limited availability of screening services for HTN/DM at the HIV CTC

Although some participants reported having their blood pressure measured routinely at the HIV CTC, the majority experienced inconsistencies in this service due to issues with malfunctioning blood pressure machines. Participants from regional referral hospitals reported a higher consistency in the provision of HTN screening services compared to those from district hospitals and health centers. One participant said,

*“…I wish it was possible to check blood pressure whenever we come to the HIV clinic…we used to do that before but later on they stopped…they told us that the machines had defaults…”* Female, 58 years, hypertensive, health center.

None of the participants, regardless of the level of the facility they were recruited from reported having their blood sugar measured at the HIV CTC. None of our participants reported undergoing screening for DM symptoms, such as polyuria, polydipsia, and polyphagia, as recommended by the guidelines. One participant said,

*“…Here, we are tested (for hypertension) every time we come, although I do not know my levels at the moment…they do check my blood pressure but they don’t test blood my blood sugar…”* Female, 34 years, hypertensive, regional referral hospital.

##### Lack of anti-hypertensives and diabetes medication at the HIV CTC

Participants reported that even when they had their BP measured at the HIV CTC and were found to have high blood pressure, they never received medication at the HIV CTC. However, participants reported being made aware of their HTN diagnosis and were advised to seek appropriate medication to manage their HTN, particularly as these medications were not being provided at the HIV CTC. One participant said,

*“…No. I don’t get here (hypertension medication) but they told me if I have a clinic, I should try to get proper medications because my hypertension is bad…”* Female, 56 years, hypertensive, regional referral hospital.

#### Accommodation

##### Perceived complexity of HTN/DM care alongside HIV care

Because of the reported poor availability of HTN/DM care services (screening and medication) at the HIV CTC and poor referral systems, participants sought care independently at other outpatient clinics within public and private health facilities. However, participants’ perceptions of healthcare services for HTN/DM indicated a characterization of complexity, lack of coordination, and inadequate tailoring to meet their needs as individuals with multiple chronic conditions including HIV infection. Participants reported facing difficulties navigating the care delivery system at the NCD clinic and poorly multiple coordinated appointment schedules that made it challenging to access and utilize HTN/DM care services in addition to HIV care. As a result, participants found care for HTN/DM time-consuming and frustrating. As he narrated his experience with services at the NCD clinic at a public health facility one participant said,

*“…You know when you fall sick, you desperately seek help… but the process is very difficult because you queue to reach the reception, then you wait to see the doctor. Afterward, you queue up to pay for the tests your doctor ordered. After testing and returning the results to your doctor, he prescribes your medications, but then, you must first go ask if they are available at the pharmacy. If they are available, you then go and queue up to pay for your prescribed medications and then go back to the pharmacy to pick up medications. So, the whole process is time-consuming, and you might waste the whole day there if you are not keen enough. That is the problem I see…”* Male, 60 years, hypertensive and diabetic, regional referral hospital.

Some participants reported that while they were keen to seek treatment for their HTN/DM, they failed to access care at the NCD clinics due to long waiting hours. One participant said:

“…*I once struggled, when I went (to the NCD clinic), I found a big queue and at that time services had not started yet. So, I sat there for a long time until I started to give up. I also struggled to find rooms to get services since I was still new and didn’t know the procedures, I spent the whole day and I eventually left. I really wanted to be going (NCD clinic) but it was not possible…”* Male, 55 years, diabetic, district hospital.

Participants also reported being frustrated with multiple uncoordinated appointment schedules as individuals in need of attending multiple clinics for their co-existing conditions. When such clinics are scheduled on different dates, patients report losing working days and incurring extra transportation costs to access the services. One patient said,

*“…Having different clinic dates is a challenge… because there are times you have to go two different clinics in one month, you use bus fare but also it becomes a challenge especially if you have other work to do…”* Female, 34 years, hypertensive, regional referral hospital.

In addition, when these clinics are scheduled on the same date, patients reported being exhausted by the need to travel back and forth between different health facilities to access the services they require for their HIV and their HTN/DM. Several participants expressed their strong preferences for receiving all the services (including care for HTN/DM) at the HIV CTC (integrated services). One participant said,

*“…Personally due to my current situation (having both HIV infection and hypertension), I was asking if it is possible for these services to be combined because sometimes you are assigned to come to the HIV clinic on the same day you have a NCD clinic, so it becomes challenging. The challenge is going back and forth and then you start a fresh queue. The process is very tiring, so when you get out of the hypertension you find yourself very tired…”* Female, 34 years, hypertensive, regional referral hospital.

Healthcare services for HTN/DM at private health facilities were perceived to be less fragmented compared to services at public health facilities. Some participants reported preferring going to private health facilities as the services are more efficient and they consequently spend less amount of time and could continue with their daily activities afterward. One participant said,

*“…There (at the private clinic), you receive all the services (related to hypertension and diabetes) you require first and then you make all payments on your way out as you receive your medications…so it is better to go there (private clinic), compared to a government center (public health facility) as you won’t have to postpone all your activities for the day…”* Male, 60 years, hypertensive and diabetic, regional referral hospital.

##### Lack of (tailored) patient education that meets ALHIV comorbidities care needs

Patient education provided was perceived not to be tailored to their needs as individuals with HTN/DM in addition to HIV infection. One participant reported being confused with contradictory information regarding the diet of someone with diabetes. While at the HIV clinic, she is told to consume plenty of any sort of fruits as individuals living with HIV infection, this information was in contradiction to the recommendations she received as a diabetic patient at the private diabetic clinic. The participant said,

*“…In fact, initially it was a dilemma to me because an HIV-positive patient needs to eat well, eat fruits thoroughly; eat good food eeh, which will give him/her good health. But now when it comes to other problems like diabetes, you are required to adhere to conditions, you are not required to eat such good food…”* Female, 42 years, diabetic, regional referral hospital.

Even though participants were made aware of their HTN diagnosis at the HIV CTCs, majority never received a formal referral to the NCD clinics. Participants sought care for HTN with minimal guidance from the providers at the HIV CTC. At the NCD clinic or any other facility HTN care was sought, participants also reported receiving inadequate guidance on HTN management from the providers at such clinics. Several participants reported being prescribed one-month supplies of HTN medication without receiving clear instructions regarding the need for ongoing medication use, follow-up visits to the health facility, and regular monitoring of their blood pressure levels. One participant said,

*“…I used the medication for a month, but when I was taking the medication, I was not told what follows after finishing the dose I just knew when you finish the dose you are done…”* Male, 67 years, hypertensive, district hospital.

To this end, participants expressed a desire for comprehensive discussions pertaining to HTN/DM management, available management options, and possible consequences of discontinuing medication use with providers after being initiated on treatment. One participant said,

*“…With hypertension…I wish there was a doctor to give us instructions on what are the consequences if you do not use medication, we have not been educated. Also, what are the consequences if you stop for a day or two, what to do? We have not been educated about high blood pressure medications. I do not go to the clinic (NCD clinic), and I don’t see any indication if there will be problems (complications) in the future…”* Female, 58 years, hypertensive, regional referral hospital.

#### Affordability

##### Unaffordable cost of HTN/DM medication

Participants reported having difficulty affording their HTN/DM medications. This cost of medication was identified as a major challenge and a primary reason for the lack of treatment continuity. Participants reported intermittently taking their HTN/DM medication to relieve their current symptoms. They would stop taking the medication immediately after the symptoms disappeared and resume treatment when the symptoms reappeared due to the inability to afford the costs of the medication. One participant said,

*“…I only seek treatment when I’m ill or if my blood sugar is high. When it gets high, it is when I start buying medication for a month, which I will use until I feel better. If I go and test and see that it has gone back to 11, I stop taking the medication. Sometimes, a month and a half can go by without getting seriously high, then it spikes up again. That is how I live…”* Male, 60 years, hypertensive and diabetic, regional referral hospital.

Consequently, participants reported worsening symptoms that significantly affected their ability to perform activities of daily living. One participant said,

*“…I had eye pain, then my head was aching especially on the face. Also, my vision was deteriorating and since I work as a tailor, I could not even knit a thread through a needle hole…”* Male, 61 years, diabetic, regional referral hospital.

The financial impact of the medication cost was milder among participants with health insurance. However, some participants reported being denied HTN/DM medications since such medications were not part of their insurance plan while unable to afford better health insurance plans. These participants had to pay out-off pocket for these medications. One participant said,

*“…It (insurance plan) doesn’t cover… at the pharmacy, they check the name of the medicines and tell you we write it for you to buy because they are very expensive and the insurance is low and the ability to get big insurance I don’t have…”* Male, 61 years, hypertensive and diabetic, district hospital.

The financial impact of the medication cost was worse among participants with additional comorbidities such as cancer and those already with HTN/DM-related complications. One participant said,

*“…I have one more dose that was added for testicular cancer, a doctor wrote a dose which cost 25,000TZS ($8)… it has been three months now I continue to use them. Therefore, if you add the cost for diabetes and blood pressure altogether it is very expensive more than 50,000TZS ($17)…”* Male, 61 years, hypertensive, diabetic, prostate cancer, district hospital.

##### Unaffordable consultation costs at HTN/DM clinic

The cost of consultations at the NCD clinic was also identified as a barrier to access care in our study population. Participants reported suspending visits to the NCD clinic because they could not afford to keep up with the consultation cost. One participant said,

*“…I had HIV and then diabetes followed. I visited the hospital when I was feeling a fever and after the checkup my blood sugar was twelve, it was when I started to go to the NCD clinic. And there (at the NCD clinic), they told me to come with 10,000TZS ($4) whenever I visit the clinic so due to my economic status, I failed…”* Male, 61 years, diabetic, regional referral hospital.

The costs for consultation were even higher when care for HTN/DM was sought at a private facility. One participant said,

*“…Cost is the biggest challenge…because a single tablet for hypertension costs 1,000TZS ($.64) and it costs 30,000TZS ($13) for 30 days. If you go to a private hospital, they will also add their profit and more cost for registration, and you will find it costing 40,000TZS ($17) or 50,000TZS ($22). If you go to the hospital, you must prepare yourself for a month’s cost. So, when you compare it to our income, it becomes too expensive, and that is exactly the problem…”* Male, 60 years, hypertensive and diabetic, regional referral hospital.

#### Acceptability

##### Fear of side effects of HTN/DM medication

Participants expressed their fear of side effects related to the use of HTN/DM medication in addition to ARTs. Specifically, participants reported experiencing physical side effects, such as dizziness and fatigue. Subsequently, some participants made the decision to discontinue the use of these medications while others opted for herbal medication as an alternative. One participant reported even being advised by the provider to stop diabetes treatment after she complained about being fatigued.

*“…The challenge was that these medications used to make me so tired… I had no energy at all. That is when they (providers) told me to stop the medication for my blood sugar and concentrate on ART…”* Female, 42 years, diabetic, district hospital.

In addition, one participant reported a sense of anxiety that was attributed to the simultaneous use of multiple medications.

*“…I keep asking myself…is it that I have died inside because of lots of serious drugs I take…that is what is in my head right now…”* Female, 45 years, hypertensive, regional referral hospital.

Participants also expressed a fear of having to take multiple medications for the rest of their lives, including HTN/DM medication along with ARTs. As a result, they reported temporarily stopping their medication use, especially when they had no noticeable symptoms.

*“…When I see that my blood pressure has stabilized, I stop, because they say we shouldn’t like to take too much medicine. I take high blood pressure medications 3 or 4 times a year. This is the 10th year I am just taking medication; I am worried because i am using 3 types of medications, there are many…”* female, 52 years, hypertensive, regional referral hospital.

##### Perceived ineffectiveness of HTN/DM medication

There was also a perception that hypertensive and/or diabetes medications are ineffective in controlling HTN/DM. Participants reported stopping using HTN/DM medication after (themselves or peers) being on treatment for a considerable duration without relief. Consequently, they stopped using the medication or combined HTN/DM medication with herbal treatment.

*“…I went to the herbal clinic in search of an alternative, hoping to get treated. Because all these medications (hypertension and diabetes medication) are just for pain relief and not curative. They (at the herbal clinic) claimed to have medications that could cure diabetes completely…”* Male, 60 years, hypertensive and diabetic, regional referral hospital.

Participants also compared HTN/DM medication to ART; stating that in contrast to the management of HIV with only ART, there are other alternatives to manage HTN/DM including resting, lifestyle modification, and the use of perceived cheaper herbal medication.

*“…I could use alternative therapies. For example, sometimes when I feel hypertensive, I turn on the TV or Radio and watch or listen to music and I feel good. Or you can chew your garlic, drink plenty of water and sleep. When you wake up in the morning you feel renewed. These HIV drugs have no alternative. I have witnessed many people who decided to stop taking HIV drugs and it was their end…”* Female, 52 years, hypertensive, regional referral hospital.

#### Accessibility

##### High transportation costs

Participants reported incurring a significant financial burden while accessing care for HTN/DM in addition to care for HIV infection at different health facilities. This was specifically a challenge among participants from health centers who were referred to higher level district hospitals and regional referral hospitals for further management at NCD clinics since health facilities do not have NCD clinics nor specialized care for HTN/DM. One participant said,

*“…I do not even have a car which I would say I would just drive. I must have 300TZS ($0.1) and 400TZS ($0.1) for the bus fare. No money to come here (to the HIV clinic) and go there (to the NCD clinic) but if I come only here (HIV clinic) I can even get a lift and just drop here. Who can give you a lift up to here and then drop you at Mwananyamala (a regional referral hospital with an NCD clinic)?…”* Female, 57 years, hypertensive, health center.

Even when NCD clinics were in the same health facility as the HIV CTC, participants reported high burden of transportation costs was attributed to the necessity of attending multiple appointments with different dates. Some participants mentioned that they stopped making regular visits to the NCD clinic even located within the same proximity of the main hospital as the HIV clinic. Instead, they opted to visit nearby lower level health facilities close to their homes to check their blood pressure and/or sugar levels. If found high, the providers at such facilities prescribed medications for the participants to purchase at community pharmacies. This approach allowed them to manage their NCDs while decreasing transportation costs. One participant said,

*“…Providers were checking and insisting that I should go and get medications because they don’t prescribe them here (at the HIV clinic). Going to the clinic (NCD clinic) is somehow hard because sometimes I don’t have transport money, so I decided to go to a facility near my home to check, they prescribe medications for me then I go to buy them…”* Female, 56 years, hypertensive, regional referral hospital.

#### Coping strategies

Participants mentioned using various strategies to cope with the financial burden of medication, consultations, and transportation. They reported frequently seeking support from family, community, and church members to afford the cost of medication and consultations. Some participants, especially younger participants focused on lifestyle modification such as engaging in physical exercise and adopting a balanced diet. Additionally, participants reported utilizing lower level health facilities and community pharmacies near the homes to monitor their blood pressure and sugar levels instead of making visits to the health facilities where they are required to pay for doctor’s consultations and transport fee. As they narrate their experiences in the process of coping with the cost of HTN/DM medications participants said,

*“…Drugs for hypertension and diabetes are too expensive. I could spend up to 60,000TZS ($26…drugs for diabetes are even more expensive. One tablet costs 1000-1500TZS ($0.45-$0.65). My children and relatives usually help me buy a few medications at a time…that is I only buy for 10,000TZs ($4) and when they are finished, I buy again for 10,000TZS”* Female, 46 years, hypertensive and diabetic, regional referral hospital.

*“…I think I do not need medication, I am keen on what I was told, to exercise and eat non-carbohydrate food. I have found it helpful and I have not thought of doing regular visits to the NCD clinic…”* male, 42 years, diabetic, district hospital.

*“…When I start experiencing symptoms I just go and check and when I find it high I go to the pharmacy…I live at Boko there is a pharmacy that checks malaria and blood pressure, so I check there and I know the medicines that I take and I go buy…”* Female, 58 years, hypertensive, health center.

However, some participants resorted to maladaptive coping strategies such as relying on perceived cheaper herbal remedies instead of pharmaceutical medications. The use of herbal remedies was more common among older participants compared to relatively younger counterparts who preferred to modify their lifestyle through healthy eating and engaging in regular physical exercise. Others opted to monitor symptoms related to HTN/DM at home. When overwhelmed with the symptoms, they reported buying small doses of medication at the community pharmacies using old prescriptions. As they narrate their experiences in the process of coping with the cost of HTN/DM medications participants said,

*“…I have decided to use mlonge (herbal medication) and not to go to the hospital because going to the hospital needs money and sometimes money is hard to get…”* Male, 75 years, hypertensive, health center.

*“…Since I have my documents (old medication prescriptions), in case I fail to go to the hospital, I can go to a pharmacy, get medications and get relief…”* male, 60 years, diabetic and hypertensive, regional referral hospital.

## Discussion

This study explored lived experiences of ALHIV and the challenges they experienced when accessing care for their comorbid HTN/DM within HIV CTCs and other healthcare settings in urban Tanzania. Our findings highlight suboptimal HTN/DM care at HIV CTCs, despite existing guidelines in Tanzania [18] on the screening, diagnosis, and management of such conditions as a strategy to prevent CVD among ALHIV. Participants reported insufficient screening of both HTN/DM, with particular emphasis on deficiency in diabetes screening. Given the frequent visits made by ALHIV to the HIV CTCs, the scarcity of screening at such clinics signifies a missed opportunity for early diagnosis and treatment initiation, especially at a time when the risk of developing irreversible complications, which are more costly to manage, is at its minimum. Early diagnosis and initiation of treatment coupled with continuous patient education on healthy lifestyles is vital for the prevention of CVD [33]. 

Leveraging a well-established HIV care platform [34] including infrastructure and human resources at HIV CTCs, offers the possibility to facilitate routine screening and consequently early diagnosis of NCD-related comorbidities [35]. Within SSA, the integration of NCD care into HIV CTCs has demonstrated success in improving clinical outcomes in prior research [36–38].. In Tanzania, although policies promote the integration of NCD services into HIV clinics [18, 39], there is limited evidence regarding the actual adoption, implementation, and effectiveness of these recommendations [40]. With the existing barriers within HIV CTCs highlighted in this study, integration without appropriate resourcing runs the risk of reducing the quality of current HIV services without improving NCD care.

However, with minimal resources, providers at HIV CTCs could be trained to provide tailored patient education for ALHIV with existing CVD-related comorbidities such as HTN/DM. In our study, participants reported receiving minimal guidance from their providers regarding the management of HTN/DM. Consequently, their understanding of both management and potential complications of poor HTN/DM management was limited which resulted in negative perceptions toward medications for HTN/DM including perceived ineffectiveness, impacting adherence, and seeking alternative therapies. The provision of patient education including through unique innovations such as mHealth has been associated with improvements in health literacy, health-seeking behavior, and treatment adherence among individuals with chronic conditions [41–43]. Participants in our study clearly indicated their readiness and desire to receive patient education tailored to managing their HTN/DM. As the burden of CVD-related comorbidities increases and the population aging with HIV expands, there is a pressing need to explore innovative approaches for providing effective patient education on multimorbidity management in this population, regardless of location. **Further research should be conducted among providers at the HIV CTC to explore other barriers and facilitators to provision of adequate tailored patient education regarding NCDs prevention and management. Possible barriers include providers lack motivation to deliver holistic care to ALHIV and HIV program’s emphasis on treatment adherence and viral suppression and not on NCD care.**

The need to access care for two or more conditions; HTN/DM in conjunction with HIV infection, either across diverse health facilities or within distinct sections of the same facility, was linked to several challenges. These included substantial strains on time while navigating a healthcare delivery system that is structured differently from that at the HIV CTCs, additional transport costs incurred during transitions between clinics, and long clinic waits, that restricted their participation in income-generating activities. Such findings indicate a high demand for integrated health services that link HIV and CVD-related care (especially HTN/DM care). Participants in our study also expressed a preference for integrated services as a potential solution to the challenges they encountered. Studies from SSA already suggest that integration is feasible and would improve quality of clinical care, and clinical and cost-effectiveness [35, 44, 45]. However, HIV CTCs in SSA, particularly in Tanzania are still overwhelmed with patients and face significant structural barriers such as shortages of equipment, staff, and space [46–49]. Further understanding of the barriers to the provision of HIV/NCD integrated care in Tanzania may facilitate the appropriate design and implementation of integration to improve cardiovascular health among ALHIV. Participants also reported relying on lower-level health facilities including dispensaries to obtain medication prescriptions and for blood pressure and sugar levels monitoring. Intensifying ongoing health system strengthening efforts in Tanzania [50] including the training of primary healthcare providers in the management of HTN/DM, could significantly aid in the prevention of NCDs.

Lack of care and treatment continuity emerged as a major challenge experienced by ALHIV with comorbid HTN/DM. Among other reasons, discontinuation of care was attributed to the high cost of medication. **While ART is provided for free, patients are required to pay out-of-pocket (if not insured by public/private health insurance) for NCD-related care including care for HTN/DM if they seek NCD-related care outside the HIV CTCs.** Care for NCD has been shown to contribute to severe financial hardships, especially among households already living in poverty, which constitutes most households in SSA [51]. Due to the high cost of medications, participants in our study opted for lifestyle modifications, including adopting healthier eating habits and engaging in regular physical exercise. Lifestyle modification is a core component of self-management behaviors for HTN/DM that are known to reduce the risk of NCD and related complications [52–54]. However, prevention of NCD complications through self-management also requires regular monitoring of blood pressure and glucose levels and often the use of medications for optimal control [52–54]. Exploration of cost-effective ways that would ensure access to CVD risk factors medication (such as pharmaceutical company assistance programs) and care continuity in this population is of paramount importance. Other coping strategies reported by our participants such as the use of herbal medicines lack scientific evidence of their effectiveness in controlling blood pressure and sugar levels. In addition, there are documented concerns about their toxicity and potential health risks [55, 56]. This further highlights an urgent need for the provision of tailored patient education on appropriate approaches to manage CVD-related comorbidities among ALHIV.

### Study strengths and limitations

Our study offers ALHIV’s perspective on the challenges faced by patients living with multiple chronic conditions including HIV in Dar es Salaam. We recruited ALHIV with HTN/DM from diverse age ranges and who had lived with the comorbid condition of HTN/DM for varying numbers of years, encompassing three different levels of health facilities, enabling us to explore a wide range of experiences, challenges, and coping strategies. In addition, we employed a well-established framework, enhancing valuable cross-study comparisons and contributing to the accumulation of knowledge. However, we had a few limitations. Firstly, our population came from six high-volume HIV CTCs in an urban setting in Tanzania. As a result, our findings could have limited generalization to HIV CTCs in other settings. We recruited ALHIV with HTN/DM who are in HIV care, potentially missing the perspectives of those in the community. Future research among ALHIV with HTN/DM not in HIV care is crucial to inform interventions promoting adherence to care, given the chronic nature of these conditions. We also did not confirm reports around access and availability of services as well as control of participants’ comorbid conditions. Also, our study did not include HIV CTC providers’ perspectives that would further strengthen recommendations for improvements from our findings.

## Conclusion

Our findings highlight challenges faced by ALHIV as they seek and access care for HTN/DM within HIV CTCs and other health facilities. Challenges encountered outside the HIV CTCs, including limited HTN/DM service accessibility and accommodation, present a potential for enhanced NCD care integration within the HIV CTCs. Expressed preference for HIV/NCD integrated care in this population, opens opportunities for researching effective integrated care models. Inadequate care for such comorbidities places ALHIV at risk of complications that could jeopardize the success of HIV programs. This impact extends to key elements of HIV care, including ART adherence and viral suppression.

### Electronic supplementary material

Below is the link to the electronic supplementary material.

Supplementary Material 1

Supplementary Material 2

Supplementary Material 3

## Abbreviations

ALHIV: Adults Living with HIV
ART: Antiretroviral Therapy
CTC: Care and Treatment Clinic
CVD: Cardiovascular Disease
DM: Diabetes Mellitus
HTN: Hypertension
NCD: Non-Communicable Diseases
SSA: Sub-Saharan Africa
