# Supplementary material for: “I only seek treatment when I am ill”: experiences of hypertension and diabetes care among adults living with HIV in urban Tanzania
Source: BMC Health Serv Res. 2024 Feb 9;24:186. doi: 10.1186/s12913-024-10688-8 (PMC10858457; doi:10.1186/s12913-024-10688-8)
Supplement: Supplementary file 1 — Supplementary Material 1 [file 12913_2024_10688_MOESM1_ESM.docx]

Semi-structured interview guide for PLHIV with CVD risk factors (HTN and/or diabetes) and on treatment – English version

Age: Sex:

Condition: Years lived with this condition:

1. Do you have any questions for us before we start?
2. We would like to know, in general how do you consider your overall health?

**Probe:**

- On the reason for the rate
- Is it all the time or there was a time he/she had a different perception or better health?

1. How do you perceive your **general health** as a patient living with HIV and hypertension or diabetes?

**Probe:**

- What aspects of your health did you think about when you answered the question?
- What are the reasons behind this perception, Did you think about your performance of your responsibilities at work or at home with your family or marriage?
- What started or what was diagnosed first? Is it HIV or hypertension or diabetes?

1. How do you perceive your **illness status** as a patient living with HIV and with hypertension or diabetes?

**Probe:**

- Do you receive care for both conditions at this clinic?
- Specifically, which service related to your hypertension or diabetes do you receive at this clinic? (care includes health education, screening, receiving a diagnosis, treatment or referral for either hypertension or diabetes)
- How do you find the service ( especially services related hypertension or diabetes care) provided at this clinic
- How satisfied are you with these services? (Think of the time spent waiting for services, number of patients, quality, privacy, providers language, availability of medications and investigations and cleanliness)

1. Would you prefer to receive these services at this clinic if they were available?

Probe:

- If yes, why do you feel that way?
- If no, why do you feel that way? Where would you prefer to receive these services

1. In your opinion, how can the care for hypertension or diabetes be improved at the CTC?
2. If not where do you receive this care?

****If they do not receive any cardiovascular related care at this clinic ask about any other clinic they go to for cardiovascular related care****

1. Generally, what is your perception on the **quality of care** for your hypertension/diabetes that you receive at that clinic?

Probe:

- Are you satisfied with the quality? (Think of the time spent waiting for services, number of patients, quality, privacy, providers language, availability of medications and investigations and cleanliness)
- Should anything change? Should the services continue as they are?

1. What has been your **experience with the care** for hypertension or diabetes that you have received at this clinic for the past 12 months?

Probe:

- What were your **expectations** during this visit to the clinic?
- What aspects of your visit to the clinic were you thinking about when you answered that question?

1. How can you describe your **adherence to treatment** for both HIV as well as hypertension/diabetes?

Probe:

- How does multi-drug use make you feel?
- How do you ensure adherence to treatment for both HIV and hypertension or diabetes?
- Do you think that medication for HIV is more important, equally important or less important than medication that you take for your hypertension or diabetes? Please tell me more about why you think of this,
- If there was an opportunity to leave medication for one condition, which condition would that be? And why?
- On average day, do you think differently about your Antiretroviral drugs compared to your hypertension or diabetes medication?

1. What is your opinion on the integration of cardiovascular related services into the HIV clinic that you are currently attending?

Probe:

- What would be the advantages?
- What would be the challenges?
- If you think there is no need for integration, why do you think so?
  - What would be the advantage?
  - What about the challenges?

1. In your opinion, how we could improve services provided at this clinic?
2. Is there any other information or other aspects about the care for hypertension or diabetes that you receive that you think would be useful for us to know?

Thanks for agreeing to take time to meet with me today

Semi-structured interview guide for PLHIV with CVD risk factors (HTN and/or diabetes) and on treatment – Swahili version

Age: Sex:

Condition: Years lived with this condition:

1. Je una swali lolote kabla hatujaanza?
2. Kwa ujumla unaweza kuzungumziaje hali yako ya afya?

Probe:

- Kwanini unazungumzia hivo hali yako?
- Je, ni kila wakati unakua na hali hiyo, je kuna kipindi ulikua na mtazamo tofauti au kuna kipindi ulikua na hali nzuri zaid?

1. Kama mtu unaeishi na maambukizi ya VVU na pressure ya juu au kisukari, unaonaje hali yako ya kiafya kwa ujumla?

Probe:

- Ni upande (vipengele) upi wa afya yako uliofikiria ukiwa unajibu swali hili?
- Kwanini unaitazama afya yako ivyo? Je umewaza jinsi ya ufanyaji wako kazi au majukumu ya nyumban, familia au ndoa?
- Kipi kilianza/kiligundulika kwanza, maambukizi ya VVU au pressure/kisukari? Kulikua na mabadiliko yapi baada ya kugundua una ugonjwa mwengine mkubwa?

1. Unaonaje hali yako ya ugonjwa/ kuugua kwa sasa kama mtu unaeishi na maambukizi ya VVU na pressure ya juu au kisukari?

Probe:

- Je, unapata huduma za magonjwa yote mawili kwenye kliniki hii?
- Kwa uhakika, ni huduma zipi hasa unapata? (Huduma hii inaweza kuwa ushauri nasihi, huduma ya uchunguzi na ugunduzi, matibabu au kupewa rufaa kwenda kituo kingine)
- Je, unazionaje huduma hizi? Dodosa zaidi juu ya huduma za pressure ya juu au kisukari
- Unaridhishwa na huduma hizi? Fikiria kuhusu muda unaotumia, ubora wa huduma, usiri, lugha ya wahudumu wa afya, usafi, upatikanaji wa dawa na vipimo

1. Je ungependa kupata huduma hiyo katika kituo hiki, kama ingekua inapatikana?

Probe:

- Kama ndio, kwanini unafikiria hivyo?
- Kama hapana, kwanini unafikiria hivo? Je ungependelea kupata wapi huduma hii?

1. Kwa maoni yako, unadhani nini kifanyike kuboresha huduma ya matibabu ya pressure ya juu au kisukari inayotolewa katika vituo vya kutoa dawa za kupunguza makali ya VVU?
2. Kama hupati huduma katika kituo hiki, ni wapi unapata huduma hiyo?

****Kwa maswali anayofuata, kama hapati huduma yoyote inayohusiana na pressure ya juu au kisukari katika kituo hiki, muulize zaidi kuhusu kliniki nyengine anayoenda kupata huduma hii****

1. Kwa ujumla, nini maoni yako juu ya ubora wa huduma za afya kwa ajili ya pressure ya juu au kisukari unazopata katika kituo hicho chengine?

Probe:

- Je unaridhishwa na huduma hizo? Fikiria kuhusu muda unaotumia kusubiri kupata huduma, ubora wa huduma, usiri, lugha ya wahudumu wa afya, usafi, upatikanaji wa dawa na vipimo
- Unazionaje huduma hizo, zibaki kama zilivyo ama kipi kibadilike?

1. Zingatia mahudhurio yako ndani ya miezi 12 iliyopita, nipe uzoefu wako (experience) na huduma ya afya uliokua unapata katika kituo hicho? (Fikiria good or bad experience)

Probe:

- Ni upande (vipengele) vya hudhurio lako uliofikiria ukiwa unajibu swali hili?
- Ni yapi yalikua matarijio yako katika mahudhurio yako?

1. Unaweza kuuelezea vipi uzingatiaji (kumeza kama inavotakiwa - adherence) wako wa dawa za matibabu kwa ajili ya kupunguza makali ya VVU na pressure ya juu au kisukari?

- Unakunywa dawa mbili kwa pamoja, unachukuliaje swala (hali) hili? Je, unachangamoto zozote?
- Unahakikisha vipi kuwa unakunywa kwa usahihi dawa zote (dawa za matibabu kwa ajili ya kupunguza makali ya VVU na pressure ya juu au kisukari)
- Je unadhani dawa za matibabu kwa ajili ya kupunguza makali ya VVU ni muhimu zaidi, zina umuhimu sawa au sio muhimu sana ukilinganisha na dawa za pressure ya juu au kisukari? Tafadhali nieleze zaidi juu ya mtazamo wako, kwanini unamtazamo huo?
- Kwa mfano kungekua na ya kupunguza, ni dawa za ugonjwa upi ungezipunguza? Kwanini una mtazamo huo?
- Nini mtazamo wako juu ya dawa za VVU ukilinganisha na dawa za pressure ya juu na kisukari ambacho unacho? (Dodosa kuhusu utofauti wa gharama, ufanyaji kazi, madhara (side effects), uwingi)

1. Nini maoni yako juu ya ujumuishwaji wa huduma za afya kwa ajili ya pressure ya juu au kisukari (unazopata katika kituo hicho chengine) katika kliniki hii ya huduma za dawa za kupunguza makali ya VVU?

Probe:

- Unadhani faida yake itakua ni nini?
- Changamoto zake je?
- Kama unadhani siziunganishwe, kwanini una mtazamo huo?
  - Je faida yake itakua nini?
  - Changamoto zake je?

1. Kwa maoni yako, tunawezaje kuboresha utoaji wa huduma za afya kwa ajili ya pressure ya juu na kisukari katika kituo hiki?
2. Kuna taarifa yoyote kuhusu huduma za afya kwa ajili a pressure ya juu na kisukari unayopata ambayo unahisi ni muhimu kunieleza?

Asante kwa kukubali kutenga mda kuzungumza na mimi leo

Semi-structured interview guide for PLHIV with CVD risk factors (HTN and/or diabetes) and not on treatment – English version

Age: Sex:

Condition: Years lived with this condition:

1. Do you have any questions for us before we start?
2. We would like to know, in general how do you consider your overall health?

**Probe:**

- On the reason for the rate
- Is it all the time or there was a time he/she had a different perception or better health?

1. How do you perceive your **general health** as a patient living with HIV and with hypertension or diabetes?

**Probe:**

- What aspects of your health did you think about when you answered the question?
- What are the reasons behind this perception, Did you think about your performance of your responsibilities at work or at home with your family or marriage?
- What started or what was diagnosed first? Is it HIV or hypertension or diabetes?

1. How do you perceive your **illness status** as a patient living with HIV and with hypertension or diabetes?

**Probe:**

- Are you on any form of treatment/management for your hypertension or diabetes? (management includes lifestyle modification – healthy eating and exercise?)
- Can you describe the care for the hypertension or diabetes that you are on (also at home)? Probe on the use of traditional medicines

If NO,

- - Why are you not in any form of management for these conditions? Including traditional treatment
  - How are you managing the hypertension or diabetes that you have? Ask more on the reasons
  - Have you ever considered starting/resuming hospital based treatment/management?
    - Would you like to receive care for your hypertension/diabetes?
    - How would you wish this care to be like/delivered?

If Yes,

- Do you receive this care at the HIV clinic?
- Specifically, which service related to your hypertension or diabetes do you receive at this clinic? (care includes health education, screening, receiving a diagnosis, treatment or referral for either hypertension or diabetes)
- How do you find the service (especially services related hypertension or diabetes care) provided at this clinic

1. Would you prefer to receive treatment (medication) or other service at this clinic if they were available?

Probe:

- If yes, why do you feel that way?
- If no, why do you feel that way? Where would you prefer to receive these services

1. If the services for the hypertension or diabetes that you have were provided at the HIV clinic, would you have been on treatment by now?
2. Do you think that care for HIV infection is more important, equally important or less important than management for your hypertension or diabetes? Please tell me more about why you think of this?
3. What is your opinion on the integration of cardiovascular related services into the HIV clinic that you are currently attending?

Probe:

- What would be the advantages?
- What would be the challenges?
- If you think there is no need for integration, why do you think so?
  - What would be the advantage?
  - What about the challenges?

1. In your opinion, how we could improve services provided at this clinic?
2. Is there any other information or other aspects about the care for hypertension or diabetes that you receive that you think would be useful for us to know?

Thanks for agreeing to take time to meet with us today…

Semi-structured interview guide for PLHIV with CVD risk factors and not on treatment – Swahili version

Age: Sex:

Condition: Years lived with this condition:

1. Je una swali lolote kabla hatujaanza?
2. Kwa ujumla unaweza kuzungumziaje hali yako ya afya?

Probe:

- Kwanini unazungumzia hivo hali yako?
- Je, ni kila wakati unakua na hali hiyo, je kuna kipindi ulikua na mtazamo tofauti au kuna kipindi ulikua na hali nzuri zaid?

1. Kama mtu unaeishi na maambukizi ya VVU na pressure ya juu au kisukari, unaonaje hali yako ya kiafya kwa ujumla?

Probe:

- Ni upande (vipengele) upi wa afya yako uliofikiria ukiwa unajibu swali hili?
- Kwanini unaitazama afya yako ivyo? Je umewaza jinsi ya ufanyaji wako kazi au majukumu ya nyumban, familia au ndoa?
- Kipi kilianza/kiligundulika kwanza, maambukizi ya VVU au pressure/kisukari? Kulikua na mabadiliko yapi baada ya kugundua una ugonjwa mwengine mkubwa?

1. Unaonaje hali yako ya ugonjwa/ kuugua kwa sasa kama mtu unaeishi na maambukizi ya VVU na pressure ya juu au kisukari?

Probe:

- Je, uko kwenye aina yoyote ya matibabu kwa ajili ya pressure ya juu au kisukari ambacho unacho (lifestyle modification – mazoezi au mabadiliko ya ulaji?)
- Tafadhali nielezee aina ya matibabu unayofanya kwa sasa (hospitali au nyumbani). Dodosa juu ya matumizi ya dawa za mitishamba

Kama hapana,

- - Kwanini hauko kwenye aina yoyote ya matibabu kwa ajili ya pressure ya juu au kisukari ambacho unacho? Hata dawa za mitishamba
- Je, unatibia vipi pressure ya juu au kisukari ambacho unacho? Dodosa juu ya sababu za aina hiyo ya matibabu/tiba
- Je, umewahi kufikiria kuanza matibabu ya hospitali?
  - Je ungependa kupata matibabu kwa ajili ya pressure ya juu au kisukari ambacho unacho?
  - Je ungependa huduma hii iweje?

Kama ndio,

- Ni huduma gani unapata hapa kituoni zinazohusiana na pressure ya juu au kisukari ambacho unacho? (Huduma hii inaweza kuwa ushauri nasihi, huduma ya uchunguzi na ugunduzi, matibabu au kupewa rufaa kwenda kituo kingine)
- Kwa uhakika, ni huduma zipi hasa unapata? (Huduma hii inaweza kuwa ushauri nasihi, huduma ya uchunguzi na ugunduzi, matibabu au kupewa rufaa kwenda kituo kingine)
- Je, unazionaje huduma hizi? Dodosa zaidi juu ya huduma za pressure ya juu au kisukari

1. Je ungependa kupata huduma zengine ambazo hupati kama kupata dawa, kama zingekua zinapatikana katika kituo hiki?

Probe:

- Kama ndio, kwanini unafikiria hivyo?
- Kama hapana, kwanini unafikiria hivo? Je ungependelea kupata wapi huduma hii?

1. Unadhani huduma hizi zingekua sehemu ya clinic yako unadhani, je ungekua umeanza matibabu?
2. Je unadhani udhibiti wa makali ya VVU ni muhimu zaidi, zina umuhimu sawa au sio muhimu sana ukilinganisha na udhibiti wa pressure ya juu au kisukari? Tafadhali nieleze zaidi juu ya hili
3. Nini maoni yako juu ya ujumuishwaji wa huduma za afya kwa ajili ya pressure ya juu au kisukari katika kliniki hii ya CTC?

Probe:

- Unadhani faida yake itakua ni nini?
- Changamoto zake je?
- Kama unadhani siziunganishwe, kwanini una mtazamo huo?
  - Unadhani faida yake itakua ni nini?
  - Changamoto zake je?

1. Nini maoni yako kwa ujumla, tunawezaje kuboresha huduma hizi?
2. Kuna taarifa yoyote kuhusu huduma za magonjwa yanayohusiana na magonjwa ya moyo unayopata ambayo unahisi nu muhimu kunieleza?

Asante kwa kukubali kutenga mda kuzungumza na mimi leo
