# Supplementary material for: “I only seek treatment when I am ill”: experiences of hypertension and diabetes care among adults living with HIV in urban Tanzania
Source: BMC Health Serv Res. 2024 Feb 9;24:186. doi: 10.1186/s12913-024-10688-8 (PMC10858457; doi:10.1186/s12913-024-10688-8)
Supplement: Supplementary file 3 — Supplementary Material 3 [file 12913_2024_10688_MOESM3_ESM.docx]

**Additional file 2: Themes, categories, and illustrative quotes on the experience of care seeking for HTN/DM and coping strategies among ALHIV in Dar-es-Salaam, 2022 (n=33)**

| **Themes** | **Categories** | ***Illustrative quote*** |
| --- | --- | --- |
| Availability | Limited availability of screening services for HTN/DM at the HIV CTC | *“…I attended here (HIV CTC) for a while without checking (blood pressure) and then they started checking…they have just stopped checking again recently but previously whenever you come, they were checking hypertension…It was every month”* Female, 61 years, hypertensive, district hospital |
|  | Lack of anti-hypertensives and diabetes medication at the HIV CTC | *"...Most of the time I check pressure here at clinic (HIV clinic) but I don’t get medicine, they say it’s out of their services therefore go and buy it…"* Female, 64 years, hypertensive and diabetic, regional referral hospital |
| Accommodation | Perceived complexity of HTN/DM care alongside HIV care | *“…We usually try our level best to go there (NCD clinic) early but the services begin at 8 am, so we spend a lot of time waiting for the services. Sometimes we are told that there is no network so the system cannot work. If the network is a problem, there should be an alternative so that we do not waste a lot of time waiting for services. This will enable us to continue with our other responsibilities…”* Female, 46 years, hypertensive and diabetic, regional referral hospital |
|  |  | *“…Sometimes you are assigned ARVs clinic and on the same day you have to go to a heart clinic so it becomes challenging. The challenge is going back and forth while start a fresh queue, get heart treatment that is tiring. When you get out of there you find yourself very tired…”* Female, 34 years, hypertensive, regional referral hospital  *“…Yes, my appointment was on 31^st^ so I have already wasted time today and then another day I will waste time, asking for permission every day at work can make me lose my job…”* Female, 46 years, diabetic, regional referral hospital |
|  | Lack of (tailored) health education that meets ALHIV comorbidities care needs | *“…Most of the times when I go to the hospital, they don’t tell me that this is hypertension and I should start going to the NCD clinic, they just give me tablets…I just use and stay home but they should have enrolled me to a specific clinic so that I come frequently. At the HIV clinic when I came, they gave me tablets (ARTs) that go and use these tablets, I could just use and stay home but they initiated a clinic for me so that I come every month...”.* Female, 61 years, hypertensive, health center  *“…It was a dilemma to me because an HIV-positive patient needs to eat well, eat fruits thoroughly; eat good food eeh, which will give him/her good health. But now when it comes to other problems like diabetes, you are required to adhere to conditions, you are not required to eat such good food. I do not know which information to follow…”.* Female, 42 years, diabetes, district hospital |
| Affordability | Unaffordable cost of HTN/DM medication | *“…I ran out of medicine due to lack of money to buy medicine since I use drugs costing 3000 TZS ($1) per day. This made me unable to afford the cost and I found myself taking herbal remedies which led to my blood sugar rising and collapsing…”* Male, 49 years, diabetic, health center  *“…Regarding diabetes, it bothers me a lot, because, when I take diabetes medication for let’s say a month…they (medication) help relieve the burning sensation in my feet and the severe pain I’ve been experiencing. But I cannot afford to continue taking these medications for two or three months, since they are expensive. Therefore, once I get relief, I stop taking these medications, until I get overwhelmed by those symptoms again…”* Male, 60 years, hypertensive and diabetic, regional referral hospital |
|  | Unaffordable consultation costs at HTN/DM clinic | *“…I was feeling fever and after the checkups diabetes was twelve, it’s where now I started to go to the container (NCD clinic at the government hospital). And there, they told me to come with 10,000TZS ($4) whenever I visit the clinic so due to the economic status that I have I failed…”* Male, 61 years, diabetic, regional referral hospital |
| Acceptability | Fear of side effects of HTN/DM medication | *"…Medicines for diabetes they make me feel dizzy, I use four pills for diabetes if I use them they disturb me. If I compare with my weight khaa! You know for the medicines of HIV I use one pill, for pressure one pill while for diabetes four pills and if I have not eaten khaa! it is a problem…"* Female, 64 years, hypertensive and diabetic, regional referral hospital  *“…You know even mlonge (herbal medication) and garlic we use because we are told that these days hospital medications are not good, so they (other patients) advised us on mlonge and garlic…”* Female, 61 years, hypertensive, health center |
|  | Perceived ineffectiveness of HTN/DM medication | *“…Kisuni medications (herbal medicine) are sold from traditional herbs shops. So I boil and drink. Because the hypertension medications I use seem not to help me, I decided to add these…”* Female, 56 years, hypertensive, regional referral hospital |
| Accessibility | High cost of transportation associated with attending multiple clinics | *“...If someone has to come to the clinic here and another date to diabetes clinic elsewhere, it costs a lot in terms of bus fare and time. You can't come here to take medicine and go somewhere else in one day…”.* Male, 42 years, diabetes, district hospital  *“…But in this city moving from one place to another requires a fare unlike at home where you can just walk sometimes, but here in Dar es Salaam it’s just money and money…”* Female, 58 years, hypertensive, health center |
| Coping strategies | Adaptive coping strategies | *“…I think I do not need medication, I am keen on what I was told, to exercise and eat non-carbohydrate food. I have found it helpful and I have not thought of doing regular visits to the NCD clinic…”* male, 42 years, diabetic, district hospital |
|  | Maladaptive coping strategies | *“…I have decided to use mlonge (herbal medication) and not to go to the hospital because going to the hospital needs money and sometimes money is hard to get…”* Male, 75 years, hypertensive, health center |
